# Effect of single-administration of d-sorbitol pretreatment on the bitterness and continued willingness to take asenapine: a randomized, single-blind, placebo-controlled, crossover trial

**DOI:** 10.1186/s12888-024-05549-x

**Published:** 2024-01-30

**Authors:** Shuhei Wada, Kunihiro Iwamoto, Hiroki Okumura, Hirotake Hida, Shuichi Hiraoka, Aya Kamei, Daisuke Mori, Kiyofumi Yamada, Masahiko Ando, Norio Ozaki, Masashi Ikeda

**Affiliations:** 1 Department of Psychiatry, Nagoya University Graduate School of Medicine, 65 Tsurumai, Showa, Nagoya, Aichi 466-8550 Japan; 2 Department of Neuropsychopharmacology and Hospital Pharmacy, Nagoya University Graduate School of Medicine, 65 Tsurumai, Showa, Nagoya, Aichi 466-8560 Japan; 3 Medical Affairs Department, Meiji Seika Pharma Co., Ltd, 2-4-16Chuo-Ku, KyobashiTokyo, 104-8002 Japan; 4 Brain and Mind Research Center, Nagoya University, 65 Tsurumai, Showa, Nagoya, Aichi 466-8550 Japan; 5 Department of Advanced Medicine, Nagoya University Hospital, 65 Tsurumai, Showa, Nagoya, Aichi 466-8550 Japan; 6 Pathophysiology of Mental Disorders, Nagoya University Graduate School of Medicine, 65 Tsurumai, Showa, Nagoya, Aichi 466-8550 Japan

**Keywords:** Asenapine, Adherence, d-sorbitol pretreatment, Schizophrenia, Bitter taste, Side effects

## Abstract

**Background:**

Asenapine has unique orally-related side effects, such as a bitter taste induced by sublingual administration, which often results in discontinuation of the medication. While the FDA has approved black-cherry-flavored asenapine, several countries have prescribed only unflavored versions. Specifically, Asians commonly report experiencing the bitterness of asenapine because they are more sensitive to bitter tastes than other ethnic groups. In this study, with the aim of improving adherence by reducing the bitterness of asenapine, we investigated the effects of d-sorbitol, which reduced the bitterness parameters of taste sensors in our previous basic study on the bitterness and continuity of asenapine among patients with schizophrenia.

**Methods:**

Twenty adult patients with schizophrenia were included in this single-blind, placebo-controlled, crossover trial. Participants rinsed their mouths with single-administration of d-sorbitol or a placebo prior to each administration of asenapine. We then conducted the questionnaires and assessed changes in the bitterness of asenapine (primary end point) and willingness to continue its use (secondary end point).

**Results:**

d-sorbitol significantly improved the bitterness of asenapine (*p* = 0.038). Although it did not significantly increase the willingness to continue asenapine (*p* = 0.180), it did show improvement over the placebo in enhancing willingness to continue, especially in patients who were not accustomed to its taste.

**Conclusion:**

Our findings indicate that single-administration of d-sorbitol significantly reduces the bitterness of asenapine. In countries where flavored asenapine is not available, this finding could benefit patients who were not accustomed to its bitter taste.

**Trial registration:**

This study was registered in the Japan Registry of Clinical Trials (jRCTs041210019) on May 14, 2021.

**Supplementary Information:**

The online version contains supplementary material available at 10.1186/s12888-024-05549-x.

## Background

Antipsychotic treatment for schizophrenia is crucial for preventing relapse and rehospitalizations and improving quality of life [1]. Even though all antipsychotic drugs have demonstrated significant efficacy for overall symptoms compared with placebo, when comparing each antipsychotic, there are no significant differences in clinical efficacy other than clozapine [2]. However, each antipsychotic drug has a unique safety profile and side effects, and adherence to antipsychotics influenced by adverse events such as metabolic side effects, extrapyramidal symptoms [3], and subjective sensations such as dysphoria, which refers to a negative and unpleasant affective state [4]. To maximize the efficacy of antipsychotic treatment, we need to optimize medication adherence by considering not only drug adverse events, but also subjective experiences and attitudes toward medication from a broader perspective [5].

Asenapine is the only sublingual antipsychotic that has been approved by the United States Food and Drug Administration (FDA), in 2009. A network meta-analysis of 32 oral antipsychotics reported that asenapine specifically affects the positive, negative, and depressive symptoms associated with schizophrenia [2]. In addition, asenapine demonstrates a favorable tolerability profile; for example, it has been shown to have modest influence on weight gain, glucose intolerance, and prolactin elevation [2, 6], as well as a low incidence of extrapyramidal side effects compared with other newer second-generation antipsychotics [7]. Although asenapine has a favorable profile, however, it also has unique oral-related side effects such as a bitter taste, which patients have to tolerate without eating or drinking for 10 min after administration to maintain its bioavailability [8]. While the FDA has approved black-cherry-flavored asenapine, several countries including Japan and countries in the European Union have prescribed only unflavored versions. Furthermore, it is not clear whether flavored formulation can reduce the bitterness of asenapine because the results of a registered randomized controlled trial comparing raspberry-flavored with unflavored asenapine have not yet been published [9, 10]. A previous study reported that 36 out of 356 patients with schizophrenia (10.1%) experienced oral hypoesthesia [11, 12], and 3 out of 46 patients with schizophrenia (6.5%) stopped taking their medication because of the bitter taste [13]. To the best of our knowledge, these reports from Asia show higher rates of oral hypoesthesia than do previous reports from outside Asia, which report oral hypoesthesia in about 25 out of 572 patients with schizophrenia (5%), and oral hypoesthesia/dysgeusia combined 13 out of 213 patients with schizophrenia (6%) [10, 14]. This may be related to the fact Asians are known to be more sensitive to bitter tastes than other ethnic groups [15]. Therefore, as is the case with other pharmaceutical preparations with a bitter taste [16], the bitterness of asenapine needs to be reduced to improve adherence.

Our previous basic research demonstrated that d-sorbitol lowered the bitterness parameters of the taste sensors [17]. d-sorbitol is widely used in oral care products and is approved as a pharmaceutical additive, which makes it easy to use practically. However, whether d-sorbitol has the same effect in patients remains uncertain. While previous studies have primarily focused on reporting the efficacy and tolerability of asenapine, this research aim was to add new evidence of improving its tolerability to enhance its efficacy. Therefore, we conducted a crossover study utilizing single-administration of d-sorbitol as the primary end point to evaluate changes in the perceived bitterness of asenapine among patients with schizophrenia. As a secondary end point, we examined changes in patients’ willingness to continue asenapine with single-administration of d -sorbitol.

## Methods

### Participants

Participants were enrolled between January 18, 2022 and January 30, 2023 at the Department of Psychiatry, Nagoya University Hospital, Nagoya, Japan. The inclusion criteria were as follows: 1) adult patients with schizophrenia or psychotic features based on the Diagnostic and Statistical Manual of Mental Disorders, 5th edition (DSM-5), regardless of sex; 2) patients using sublingual asenapine tablets for more than 2 weeks; and 3) patients who could provide written informed consent. The exclusion criteria were as follows: 1) patients with physical diseases or symptoms requiring medical treatment; 2) pregnant and lactating women; 3) patients with a history of convulsions; 4) patients with a substance use disorder; 5) patients with a taste disorder; 6) patients with an olfactory disorder; 7) patients who could not understand the questionnaire protocol; and 8) other patients whose inclusion in the study was deemed inappropriate by the attending physician. The reason for excluding those with olfactory disorder is that an association between olfactory and bitter taste receptors has been suggested, and olfactory disorder may also affect the evaluation of taste [18]. Whether to exclude or discontinue any given participant was determined according to the study protocol [19] and the sample size was set as 20. This study was approved by the Nagoya University Clinical Research Review Board (CRB4180004), and written informed consent was obtained from all enrolled patients before the study began. This study was conducted in accordance with the Declaration of Helsinki.

### Study design

This study was designed as a single-blind, placebo-controlled, crossover trial, and randomization was determined using the envelope method. Placebo and d-sorbitol were administered in clear containers, as they were visually indistinguishable. The participants received d-sorbitol solution as the active drug and distilled water as the placebo. Even if the participants could distinguish between the two liquids based sweetness levels, they did not indicate which one was the placebo. Only the participants were blinded. The participants rinsed and spitted out 25 mL of the single administration of study drug immediately before taking the sublingual asenapine tablet. During the study period, the patients were instructed to rinse their mouth by themselves at home or in the hospital ward, and then to answer a multiple-choice questionnaire. This study was registered in the Japan Registry of Clinical Trials (jRCTs041210019) on May 14, 2021, and the study protocol and detailed methods have been published [19].

### Experimental schedule

**Fig. 1:**
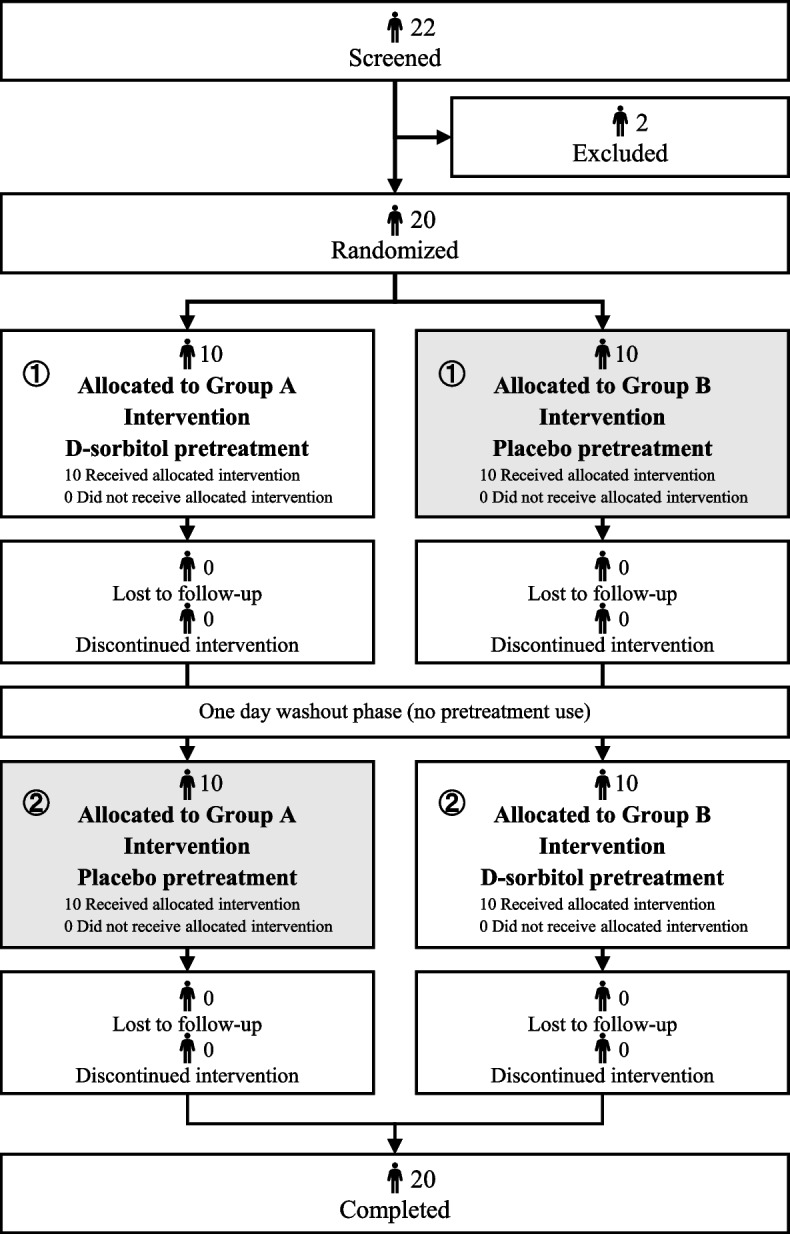
Experimental schedule and flow diagram. The participants rinsed and spitted out 25 mL of the study drug (d-sorbitol or placebo) once a day, just before the same timing of either of the two dosing times for asenapine. All participants were randomly assigned to Group A or Group B (*n* = 10 each)

The experimental schedule and flow diagram are shown in Fig. 1. The participants were divided into two groups (Groups A and B). “Group A received d-sorbitol first (test period ① using d-sorbitol and test period ② using placebo solution), and Group B received the placebo solution first (test period ① using placebo solution and test period ② using d-sorbitol). There was a 1-day interval between study periods ① and ②. This study was conducted in the ward for inpatients and at home for outpatients at the same time of day as the administration of the sublingual asenapine tablets. At the beginning of the study, baseline characteristics were obtained from all participants” [19]. After each intervention, the participants were asked to answer the post-implementation questionnaires to evaluate the outcome.

### Baseline survey content

At the beginning of the study, baseline information was obtained for the following items: “disease onset (single episode or multiple episodes), duration of illness from first onset, DSM-5 specific terms, total Positive and Negative Syndrome Scale (PANSS) score at enrollment, asenapine starting date, asenapine dosage, concomitant medications, comorbidities, smoking history, height, weight, how long it took to become accustomed to taking asenapine, duration for which the patient has considered themselves to be accustomed to the taste of asenapine, and adverse events associated with asenapine use” [19].

### Questionnaire survey content

The post-implementation questionnaires asked for choice-based Likert scale statements regarding the following:

1. Oral condition

Participants chose an answer on a scale from ① to ⑤ to describe their oral condition prior to each rinse with d-sorbitol and placebo: ①Very dry, ② Dry, ③ Normal, ④ Some saliva, ⑤ A lot of saliva.

2. Change in bitter taste when taking asenapine or placebo

“Participants chose an answer on a scale from ① to ⑦ to evaluate the bitterness of the sublingual tablet compared with the bitterness they used to feel: ① Almost no bitterness, ② Bitterness has been reduced, ③ Bitterness seems to have decreased a little, ④ No change, ⑤ Bitterness has become a little stronger, ⑥ Bitterness has become stronger, ⑦ Bitterness has become so strong that it is difficult to use” [19].

3. Whether the taste of asenapine with the intervention results in ease of continued use

Participants chose an answer on a scale from ① to ⑤ to evaluate continuity: ① Taste allows continued use without difficulty, ② Taste makes it easier to continue use compared with before, ③ Same taste as before, ④ Taste makes it more difficult to continue use compared with before, ⑤ Taste makes it difficult to continue use.

All questionnaires were conducted in Japanese.

### Study outcome

The primary end point was the taste evaluation of bitterness, which addressed post-implementation question 2. The participants self-assessed items regarding the use of single-administration of d-sorbitol and the reduction in perceived bitter taste on a 7-point scale. The secondary endpoint was the willingness to continue asenapine, which was subjectively self-rated on a 5-point scale; this addressed post-implementation question 3.

### Statistical analysis

Descriptive analyses were performed on baseline variables using the mean (standard deviation), median (interquartile range), or proportions and percentages as appropriate. To evaluate the primary and secondary endpoint, we used a 2-point scale (improved/non-improved). In the seven or five-point scale questionnaire, responses indicating “no change “and “worsening” were categorized as non-improvement. The statistical method was determined and reported in our protocol study before this research [19]. “The primary endpoint was evaluated on a 7-point Likert scale, with 1–3 points defined as improvement and 4–7 points as non-improvement. The secondary endpoint was evaluated on a 5-point Likert scale, with 1–2 points defined as improvement and 3–5 points as non-improvement. The difference in population proportions for improved/non-improved binary data was analyzed using McNemar’s test” [19]. Furthermore, post hoc analysis was performed to compare the continuity of asenapine between d-sorbitol and placebo by Fisher’s exact test in the two groups, divided by whether they had become accustomed to the taste of asenapine. Statistical analyses were conducted using R version 4.3.0 (R Foundation for Statistical Computing).

## Results

### Characteristics of the patients

We recruited 22 patients in total, among whom, 20 completed the treatment phase and were analyzed. Two patients were excluded based on the exclusion criteria (patients who could not understand the questionnaire protocol). The characteristics of the participants are shown in Table 1. All the participants were Asian people. About 19 out of 20 participants (95%), within 1 month of initial use, got accustomed to the proper usage of asenapine, for example, putting sublingual tablets under the tongue and tolerating without eating and drinking for 10 min after administration. However, at the study entry, 8 out of 20 participants (40%) had still not become accustomed to the taste of asenapine. After starting asenapine, the only increase seen in adverse events involved reports of menstrual irregularities.

**Table 1:** Baseline clinical data of the study participants with schizophrenia

Participants	*N* = 20
Age (y)	48.2 ± 13.6
Male sex	*N* = 6, 30%
PANSS total score (mean ± SD)	47.4 ± 12.3
Multiple episodes	*N* = 19, 95%
Duration of illness (y)	N (%)
< 5	2 (10%)
≧ 5 and < 10	1 (5%)
≧ 10 and < 20	7 (35%)
≧ 20	10 (50%)
Median duration of asenapine use, years (quartile)	3.7 (2.1**–**4.3)
At least one year use, n (%)	17 (85%)
Mean dose of CP equivalent, mg (quartile)	200 (200**–**400)
Concomitant medications, n (%)	
Benzodiazepine	10 (50%)
First-generation antipsychotics	0 (0%)
Second-generation antipsychotics	8 (40%)
Antidepressants	10 (50%)
Mood stabilizer	3 (15%)
Drugs for physical disorders	9 (45%)
Smoker	3 (15%)
Median BMI (quartile)	24.1 (20.6**–**28.2)
Same oral condition at the time of each intervention, n (%)	16 (80%)

*BMI* Body Mass Index, *CP* Chlorpromazine, *PANSS* Positive and Negative Syndrome Scale *SD* Standard deviation

### Primary and secondary outcomes

**Fig. 2:**
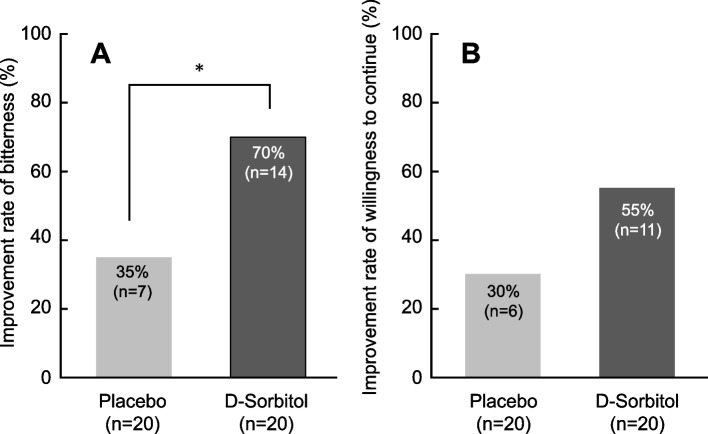
Comparison of effects with single-administration of d-sorbitol and placebo on the bitterness of asenapine and willingness to continue. *McNemar’s test, *p* < 0.05. **A** Improvement in perceived bitterness of asenapine. Improvement was defined as a score ranging from 1 to 3points on a 7-point Likert scale. **B** Improvement in willingness to continue asenapine. Improvement was defined as a score ranging from 1 to 2 points on a 5-point Likert scale

Regarding the primary outcome, we observed a significant difference in the proportions of improvements in the perceived bitterness of asenapine between single-administration of d-sorbitol and placebo (70% vs. 35%, respectively; *p* = 0.038, Cohen’s g = 0.39 [95% confidence interval (CI): 0.07, 0.48]) (shown in Fig. 2A).

However, regarding the secondary outcome, no significant differences in the proportions of willingness to continue use between single-administration of d-sorbitol and placebo were observed (55% vs. 30%, respectively; *p* = 0.180, Cohen’s g = 0.28 [95%CI: –0.05, 0.44]) (Fig. 2B).

### Subgroup analysis

**Fig. 3:**
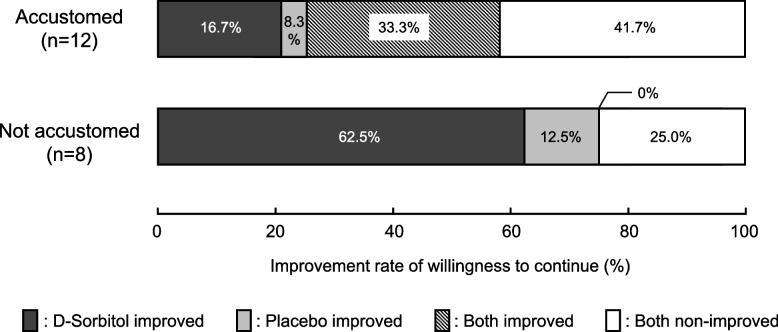
Effect of single-administration of d-sorbitol or placebo on willingness to continue asenapine between the two groups based on whether or not they had become accustomed to the taste of asenapine

As a subgroup analysis, we divided the participants into two groups depending on whether they had or had not become accustomed to the taste of asenapine. Improvement of willingness to continue occurred in 2 of 12 patients (12.5%) in accustomed group and in 5 of 8 patients (62.5%) in not accustomed group (*p* = 0.062, Cramér’s V = 0.42 [0.00, 0.96]) (Fig. 3).

## Discussion

To the best of our knowledge, this is the first intervention study focusing on the bitter taste of asenapine in patients with schizophrenia. In this single-blind, placebo-controlled, crossover trial, single-administration of d-sorbitol pretreatment significantly improved the perceived bitterness of asenapine with a large effect size [20]. However, it did not significantly enhance the willingness to continue asenapine. While not statistically significant, there was a trend for d-sorbitol to increase the rate of improvement in willingness to continue asenapine over placebo in patients who were not accustomed to the taste of asenapine.

In the present study, we demonstrated that single-administration of d-sorbitol improved the bitterness of asenapine in patients with schizophrenia. d-sorbitol has several advantages such as its low cost (JPY 25.5 per 25 mL, equivalent to USD 0.25 per 25 mL) [21, 22], its prescription availability, and its guaranteed quality assurance, and can be used without concerns about drug interactions or side effects. Even though sorbitol can cause diarrhea, just gargling the d-sorbitol can have little effect on bowel activity [23]. Furthermore, it is known that sorbitol can have a protective effect on dental cavities [24]. Alleviating the bitter taste of asenapine could significantly improve the quality of life for patients who must endure its bitter taste because taste perception is an important part of human life and drug-induced dysgeusia has a negative influence on a patient’s quality of life [25].

Regarding the secondary outcome, no significant differences in the proportions of participants reporting willingness to continue were found between single-administration of d-sorbitol and placebo. It should be noted that 40% of the patients had not become accustomed to the taste of asenapine, although 85% of participants had already taken it for at least 1 year. Because these participants might originally have been willing to continue asenapine, it is possible that there may have been little change in their willingness to continue after the addition of d-sorbitol. Nevertheless, d-sorbitol showed the potential to increase the willingness to continue asenapine in patients who were not accustomed to the taste of asenapine and may therefore be useful in patients initiating treatment on the drug. Another point influencing the implications of willingness to continue should be noted. Medication adherence is simultaneously influenced by several factors, such as socioeconomic factors, health-care teams/systems, the characteristics of the disease, disease therapies, and patient-related factors [26]. Particularly, for adults with psychiatric conditions, patient- and medication-related factors such as cognitive factors and side effects have been shown to have the greatest influenced on adherence [27]. In the present study, the participants’ willingness to continue may have been influenced by not only the degree of improvement in perceived bitterness, but also other factors such as their pathology, impaired insights into illness [28], and subjective sensations other than bitterness [4].

The results of this study showed that 40% of patients had not become accustomed to the taste of asenapine, even though they had taken it for a median duration of 3.7 years. Although the relationship between the side effects of asenapine and the reason for not being accustomed to the taste remains uncertain, our findings showed values that exceeded the previously reported rates of oral-related adverse effects [12, 29]. This discrepancy might be explained by the fact that the patients in this study may have been more comfortable reporting asenapine-related adverse events through questionnaires than during a medical examination. In addition, it might be explained by the unique focus of this study on the bitterness of asenapine because clinicians may underestimate problems related to the taste of asenapine since perception of the taste typically disappears within 1 h [30]. Furthermore, there may be differences in the strength of the perception of bitterness depending on individuals or ethnic groups. Individual differences in sensitivity to the bitter compound 6-n-propylthiouracil (PROP), a marker for taste perception, have been reported [31]. Compared with populations of European descent, Asians have a significantly higher rate of PROP supertasters who perceive it as extremely bitter [15]. Another study reported that Asians are far more sensitive to bitter tastes than individuals of African-American, white, or Hispanic ethnic origin [32]. Furthermore, clinical data have also shown a higher incidence of oral hypoesthesia reported in Asian populations (about 10%) than in Western populations (about 5%) [10, 11, 14, 29]. Therefore, clinicians should pay more attention to how patients feel about taking medicines and their side effects and consider discussing these issues with their patients and providing interventions to improve medication continuation.

This study has several limitations. First, a potential sampling bias should be noted, as the majority of our patients had been taking asenapine for an extended period of time. Different results may be obtained in new users who have not become accustomed to the taste of asenapine. Second, we calculated the sample size depending based only on the change in perceived bitterness when patients had started asenapine for 4 weeks. We could not estimate the effect on continuity because this was the first intervention trial to address the bitterness of asenapine. Therefore, the sample size might be insufficient for accurately evaluating the change of willingness to continue asenapine. Third, participants are thought to judge their willingness to continue based on not only the improvement in perceived bitterness, but also other factors such as inconvenience with using d-sorbitol pretreatment. It would be beneficial to confirm the reason for their answer and whether they answered depending on the change in perceived bitterness. Fourth, we did not perform sex-based analyses in this trial because of the large difference in the sex ratio. Fifth, the observational period was only 3 days. A longer follow-up period is needed to establish the effect of d-sorbitol on adherence, even though the participants answered that d-sorbitol could help them continue asenapine. Sixth, the phrase “not accustomed to the taste” for a patient may give not only a negative impression, but also a positive or neutral impression. The impact of that phrase on the outcome needs to be considered.

## Conclusion

The results of this single-blind, placebo-controlled, crossover trial indicated that single-administration of d-sorbitol pretreatment significantly improved the perceived bitterness of asenapine compared with placebo among patients with schizophrenia. In countries where flavored asenapine is not available, this finding could benefit patients who find the bitterness of asenapine challenging. Furthermore, d-sorbitol pretreatment may improve patients’ quality of life and adherence to asenapine, especially those who have difficulty tolerating the bitter taste of asenapine.

### Supplementary Information

**Additional file 1.** Questionnaire survey content.

## Data Availability

The datasets generated and/or analyzed during the present study are not publicly available because data sharing was not included in the consent form but are available from the corresponding author upon reasonable request.

## Abbreviations

DSM-5: Diagnostic and Statistical Manual of Mental Disorders, 5th edition
FDA: Food and Drug Administration
PANSS: Positive and Negative Syndrome Scale
PROP: 6-N-propylthiouracil

## References

[1] Ceraso A, Lin JJ, Schneider-Thoma J, Siafis S, Heres S, Kissling W, Davis JM, Leucht S (2022). Maintenance treatment with antipsychotic drugs in schizophrenia: a cochrane systematic review and meta-analysis. Schizophr Bull.

[2] Huhn M, Nikolakopoulou A, Schneider-Thoma J, Krause M, Samara M, Peter N, Arndt T, Bäckers L, Rothe P, Cipriani A (2019). Comparative efficacy and tolerability of 32 oral antipsychotics for the acute treatment of adults with multi-episode schizophrenia: a systematic review and network meta-analysis. Lancet.

[3] Dibonaventura M, Gabriel S, Dupclay L, Gupta S, Kim E (2012). A patient perspective of the impact of medication side effects on adherence: results of a cross-sectional nationwide survey of patients with schizophrenia. BMC Psychiatry.

[4] Wu HE, Okusaga OO (2015). Antipsychotic medication-induced dysphoria: its meaning, association with typical vs. Atypical medications and impact on adherence. Psychiatric Quart.

[5] Awad AG (2019). Revisiting the concept of subjective tolerability to antipsychotic medications in schizophrenia and its clinical and research implications: 30 years later. CNS Drugs.

[6] Greger J, Aladeen T, Lewandowski E, Wojcik R, Westphal E, Rainka M, Capote H (2021). Comparison of the metabolic characteristics of newer second generation Antipsychotics: Brexpiprazole, Lurasidone, Asenapine, Cariprazine, and Iloperidone with Olanzapine as a comparator. J Clin Psychopharmacol.

[7] Chow CL, Kadouh NK, Bostwick JR, Vandenberg AM (2020). Akathisia and newer second-generation antipsychotic drugs: a review of current evidence. Pharmacother J Hum Pharmacol Drug Ther.

[8] Citrome L (2014). Asenapine review, part I: chemistry, receptor affinity profile, pharmacokinetics and metabolism. Exp Opin Drug Metab Toxicol.

[9] Citrome L (2014). Asenapine review, part II: clinical efficacy, safety and tolerability. Exp Opin Drug Safety.

[10] Citrome L (2009). Asenapine for schizophrenia and bipolar disorder: a review of the efficacy and safety profile for this newly approved sublingually absorbed second-generation antipsychotic. Int J Clin Pract.

[11] Kishi T, Iwama Y, Sasagawa Y, Hiraoka S, Kamei A, Iwata N (2023). Asenapine add-on treatment for schizophrenia adults who received antipsychotics: a 52-week, open-label study. Psychiatry Clin Neurosci.

[12] Kinoshita T, Bai Y-M, Kim J-H, Miyake M, Oshima N (2016). Efficacy and safety of asenapine in Asian patients with an acute exacerbation of schizophrenia: a multicentre, randomized, double-blind, 6-week, placebo-controlled study. Psychopharmacology.

[13] Matsuzaki H, Hatano M, Iwata M, Yamada S (2021). Treatment continuation of Asenapine or Olanzapine in Japanese schizophrenia patients: a propensity score matched study. Neuropsychiatr Dis Treat.

[14] Landbloom R, Mackle M, Wu X, Kelly L, Snow-Adami L, Mcintyre RS, Mathews M, Hundt C (2017). Asenapine for the treatment of adults with an acute exacerbation of schizophrenia: results from a randomized, double-blind, fixed-dose, placebo-controlled trial with olanzapine as an active control. CNS Spectr.

[15] Yang Q, Williamson A-M, Hasted A, Hort J (2020). Exploring the relationships between taste phenotypes, genotypes, ethnicity, gender and taste perception using Chi-square and regression tree analysis. Food Qual Prefer.

[16] Beltrán LR, Sterneder S, Hasural A, Paetz S, Hans J, Ley JP, Somoza V (2022). Reducing the bitter taste of pharmaceuticals using cell-based identification of bitter-masking compounds. Pharmaceuticals.

[17] Kaneshige J, Kon M, Kai N, Hiraoka S, Ohta M, Ozaki N (2020). Evaluation of taste-masking effect of asenapine maleate using ingredients for oral care products by taste sensor. J New Remedies Clin.

[18] Alfonso-Prieto M (2021). Bitter taste and olfactory receptors: beyond chemical sensing in the tongue and the nose. J Membr Biol.

[19] Wada S, Iwamoto K, Okumura H, Hida H, Hiraoka S, Kamei A, Mori D, Yamada K, Ozaki N (2023). Sensory evaluation of the bitterness of asenapine using D-sorbitol pretreatment: single-blind, placebo-controlled, crossover trial. BMC Psychiatry.

[20] Cohen J (1988). Tatistical power analysis for the behavioral sciences.

[21] D-sorbitol [https://www.kegg.jp/medicus-bin/similar_product?jtc=7990&route=o&column=price&refresh_button=%E6%9B%B4%E6%96%B0&japic_code=&kegg_drug=D00096&member=]

[22] Mannitol/sorbitol topical Prices, Coupons, Copay & Patient Assistance - Drugs.com [https://www.drugs.com/price-guide/mannitol-sorbitol-topical]

[23] Bauditz J, Norman K, Biering H, Lochs H, Pirlich M (2008). Severe weight loss caused by chewing gum. BMJ.

[24] Hayes C (2001). The effect of non-cariogenic sweeteners on the prevention of dental caries: a review of the evidence. J Dent Educ.

[25] Naik BS, Shetty N, Maben EV (2010). Drug-induced taste disorders. Eur J Intern Med.

[26] Adherence to long-term therapies : evidence for action. World Health Organization. [https://apps.who.int/iris/handle/10665/42682]

[27] Peh KQE, Kwan YH, Goh H, Ramchandani H, Phang JK, Lim ZY, Loh DHF, Østbye T, Blalock DV, Yoon S (2021). An adaptable framework for factors contributing to medication adherence: results from a systematic review of 102 conceptual frameworks. J Gen Intern Med.

[28] Kim J, Ozzoude M, Nakajima S, Shah P, Caravaggio F, Iwata Y, De Luca V, Graff-Guerrero A, Gerretsen P (2020). Insight and medication adherence in schizophrenia: An analysis of the CATIE trial. Neuropharmacology.

[29] Kinoshita T, Takekita Y, Hiraoka S, Tamura F, Iwama Y (2023). Long-term safety and efficacy of sublingual asenapine for the treatment of schizophrenia: A phase III extension study with follow-up for 52 weeks (P06125)-Secondary publication. Neuropsychopharmacol Rep..

[30] Musselman M, Faden J, Citrome L (2021). Asenapine: an atypical antipsychotic with atypical formulations. Ther Adv Psychopharmacol.

[31] Lim J, Urban L, Green BG (2008). Measures of individual differences in taste and creaminess perception. Chem Senses.

[32] Choi SE, Chan J (2015). Relationship of 6-n-propylthiouracil taste intensity and chili pepper use with body mass index, energy intake, and fat intake within an ethnically diverse population. J Acad Nutr Diet.

