# Supplementary material for: Effect of single-administration of d-sorbitol pretreatment on the bitterness and continued willingness to take asenapine: a randomized, single-blind, placebo-controlled, crossover trial
Source: BMC Psychiatry. 2024 Jan 30;24:81. doi: 10.1186/s12888-024-05549-x (PMC10829201; doi:10.1186/s12888-024-05549-x)
Supplement: Supplementary file 1 — Additional file 1. Questionnaire survey content. [file 12888_2024_5549_MOESM1_ESM.docx]

Questionnaire survey content

The post-implementation questionnaires asked for choice-based Likert scale statements regarding the following:

1. Oral condition

Participants chose an answer on a scale from ① to ⑤ to describe their oral condition prior to each rinse with D-sorbitol and placebo: ① Very dry, ② Dry, ③ Normal, ④ Some saliva, ⑤ A lot of saliva.

2. Change in bitter taste when taking asenapine or placebo

Participants chose an answer on a scale from ① to ⑦ to assess the bitterness of the sublingual tablet relative to their previous experiences: ① Almost no bitterness, ② Reduced bitterness, ③ Slight decrease in bitterness, ④ No change, ⑤ Slight increase in bitterness, ⑥ Increased bitterness, ⑦ Bitterness increased to a level that it is difficult to use.

3. Whether the taste of asenapine with the intervention results in ease of continued use

Participants chose an answer on a scale from ① to ⑤ to evaluate continuity: ① Taste allows continued use without difficulty, ② Taste makes it easier to continue use compared with before, ③ Same taste as before, ④ Taste makes it more difficult to continue use compared with before, ⑤ Taste makes it difficult to continue use.

All questionnaires were conducted in Japanese.
